# Association of COVID-19 vaccines and antibody response in individuals with prior Coronavirus infection

**DOI:** 10.1038/s41598-026-42177-9

**Published:** 2026-03-13

**Authors:** R. Malvika Shyamkumar, Mridula Madiyal, Geeta Bhuvanagiri, Marushka Ronita Pinto, Prajna Nayak, Aditi Chopra

**Affiliations:** 1 Department of Periodontology, Manipal College of Dental Sciences, Manipal Academy of Higher Education, Manipal, India; 2 Department of Microbiology, Kasturba Medical College, Manipal Academy of Higher Education, Manipal, India; 3 Present Address: Postgraduate Periodontics and Implant Surgery, UCLA School of Dentistry, Los Angeles, CA USA; 4 Department of Public Health Dentistry, Manipal College of Dental Sciences, Manipal Academy of Higher Education, Manipal, India

**Keywords:** Coronavirus, COVID-19, Vaccination, Pandemic, India, Covishield, Covaxin, Immunity, Diseases, Immunology, Medical research, Microbiology

## Abstract

Severe Acute Respiratory Syndrome Coronavirus-2 (SARS-CoV-2) was one of the worst pandemics and viral infections affecting humans across the globe. Many non-pharmaceutical and pharmacological interventions were initiated to prevent the spread of infection and control the disease transmission. Covishield and Covaxin were among the most common vaccines given in India to control the spread of the coronavirus and its variants among the public. To evaluate the efficacy of COVID-19 vaccines in increasing serum and salivary IgA antibody levels and how they are correlated to patients’ demographics, medical conditions, and previous history of coronavirus infection in the Udupi district, Karnataka, India. 127 participants who received two doses of the COVID-19 vaccine were recruited. Anti-SARS-CoV-2 IgA antibodies specific to COVID-19 in serum and saliva were measured by ELISA. The mean serum IgA levels were compared at 0–6 months, 6–12 months, and > 12 months. The IgA levels were compared with age, gender, history of COVID-19 infection, timing of vaccination, body mass index, and comorbidities. The mean serum IgA levels in individuals with a history of COVID-19 (12.59 ± 5.67 μg/ml) were higher than those without a history of infection (8.5 ± 7.20 μg/ml). Among those with a history of COVID-19 infection, 8.1% were infected before the vaccination, and 91.9% were infected post-vaccination. Serum IgA levels were lower in participants under 30 years of age (5.27 ± 3.14 μg/mL) compared to participants above 30 years of age (8.93 ± 4.56 μg/mL) (P = 0.001). Antibody levels were influenced by age, presence of comorbidities, and history of coronavirus infection. Individuals with prior COVID-19 infection showed higher serum IgA antibody levels. Serum and salivary IgA levels were even detected in a group of participants with more than 12 months post-vaccination period.

**Supplementary Information:**

The online version contains supplementary material available at 10.1038/s41598-026-42177-9.

## Introduction

The Coronavirus pandemic of 2019 (COVID-19) was one of the most devastating events of the twenty-first century. It was caused by a single-stranded RNA beta Coronavirus, commonly referred to as Severe Acute Respiratory Syndrome Coronavirus-2 (SARS-CoV-2)^1,2^. In December 2019, COVID-19 was first reported in China^3–5^. According to the World Health Organization (WHO), as of 13^th^ April 2024, more than 704.8 million people were infected, and more than 7.01 million people died because of COVID-19^5^. Among all the countries affected by COVID-19, India was the second most affected nation after the United States. According to WHO statistics, more than 77.6 million people were infected with COVID-19 in India by 2024^5^.

Many pharmacological and non-pharmacological interventions were started to control the spread of infection. Strict infection control protocols, including hand hygiene, masking, isolation, quarantine, lockdown, and social distancing, were implemented to limit the spread of infection^6–9^. Along with these non-pharmaceutical interventions, a rapid surge in the development and administration of COVID-19 vaccines was initiated to curb the pandemic. Some of the common vaccines developed against the SARS-CoV-2 virus were the Pfizer-BioNTech (Comirnaty) by Pfizer; Vaxzevria by AstraZeneca; Moderna Biotech (Spikevax) by Moderna Tx Inc.; Covovax by Novavax; Covaxin by Bharat Biotech; Covishield by the Serum Institute of India; and Sputnik V by the Gamaleya National Centre^10^. The vaccination campaign against SARS-CoV-2 in India began with the administration of two types of vaccines: Covaxin and Covishield^10,11^. A total of 13.3 million vaccines were administered in India by April 29, 2023^5^. The vaccines were given to increase the levels of immunoglobulins (Ig) in the body, provide systemic/mucosal protection, and prevent disease transmission.

IgM, IgG, and IgA are among the most common antibodies produced after natural infection and vaccination. IgM antibodies are the first to appear in the body against any viral antigen. They can be detected as early as three days after the onset of symptoms, and later they class-switch to IgG or IgA antibodies. IgA and secretory IgA (sIgA, IgA secreted in saliva) provide the first line of mucosal defense and innate immunity in the nasal, oral, digestive, and respiratory tracts^7,8,12^. sIgA is the key Ig and defense mechanism at the mucosal surfaces, as it helps to neutralize the virus at the point of entry. IgA also prevents adherence and invasion of viral particles into epithelial cells and precludes their invasion into the systemic circulation. Studies have found that IgA appears earlier than IgG after infection and can persist for months at the mucosal sites^11,13,14^. In many individuals, IgA is the primary early neutralizing antibody after COVID-19 infection and provides the frontline protection during initial exposure. SARS-CoV-2–specific IgA antibodies appear very early after infection, often before IgG. It binds strongly to the spike protein of SARS-CoV-2, blocking the virus from interacting with ACE2 receptors and lowering the risk of systemic infection^7^. Several studies have reported that higher mucosal sIgA levels are associated with lower viral loads, decreased viral shedding, and reduced transmission rates. This emphasizes the role IgA in controlling the spread of viral infection in the community^7–16^. The amount of IgA in saliva is also suggested to be an early marker for assessing the immune response following infection or vaccination^7–12,15,16^.

Various studies have tested the efficacy of vaccines in increasing serum antibody levels (anti-SARS-CoV-2 IgG, IgA, or IgM) and have reported mixed results^10,12,15^. Levin et al. (2021) found that six months after the second dose of Pfizer’s vaccine, the humoral response decreased significantly in elderly and immunocompromised individuals^12^. Another study by Wisnewski et al. (2021) found that the vaccine-specific IgG and IgA antibody levels increase immediately after vaccination with a decline after a few months^16^. Covishield and Covaxin vaccines have also been found to be effective in increasing antibody levels post-vaccination, with Covishield being more effective than Covaxin^11^. Previous studies have even tested the levels of antibodies (IgM, IgG, IgA, and total Igs) after natural COVID-19 infection and reported that the serum and salivary IgG antibodies to SARS-CoV-2 are detected in serum and saliva of all patients within 14 days of acquiring infection^13,14,17^. The Ig levels were maintained in the majority of COVID-19 patients for at least 3 months after the onset of symptoms^8,13,14,17,18^. However, there is a paucity of evidence regarding the antibody response and levels of IgA in saliva and serum in patients post vaccination in the Indian sub-continent with and without a previous history of COVID-19 infection. The WHO also stated that "it is still unclear and difficult to confirm the best time to administer a third (booster) dose of COVID-19 vaccine. The timing of a booster dose depends on the existing antibody levels after natural infection, patient-related factors, and the persistence of passive immunization in individuals achieved through vaccination”. Additionally, to our knowledge, no study has evaluated and compared the change in serum and saliva IgA antibody levels following vaccination and their comparison to patients’ demographic and previous history of COVID-19 in the Indian population. It is also unknown whether serum and salivary IgA antibodies are higher or lower in individuals with a previous history of COVID-19 infection compared to those without a history of COVID-19 infection in the Indian population. The effect of age, gender, comorbidities, oral hygiene status (OHI), and body mass index (BMI) on post-vaccination antibody levels has not been studied in the Indian population.

Therefore, the present study aims to evaluate the IgA levels in the serum and saliva after COVID-19 vaccination with the following objectives:

1. To estimate and compare mean serum and salivary IgA antibody levels in participants who received two doses of the COVID-19 vaccines.
2. To estimate and compare mean serum and salivary IgA antibody levels in participants who received the COVID-19 vaccines with and without a previous history of COVID-19 infection.
3. To evaluate and compare the association of mean serum and salivary IgA antibody levels with age, gender, smoking status, presence of any systemic disease, oral hygiene status, history of COVID-19 infection, and comorbidities.

## Methods

The study was designed as a cross-sectional observational study. The study was conducted at the Department of Periodontology, Manipal College of Dental Sciences, Manipal, India, in collaboration with the Clinical Hematology Laboratory, Kasturba Medical Hospital, Manipal, and the Department of Microbiology, Kasturba Medical Hospital, Manipal, India. The study process was initiated from January 2022 to September 2022, following the tenets of the Declaration of Helsinki (as revised in 2013), STROBE, and Sex and Gender Equity in Research guidelines. The study was initiated after approval by the Institutional Review Committee of Kasturba Medical College and Kasturba Hospital (IEC No: 23/2022) and registration with the Clinical Trials Registry, India under registration number: CTRI/2022/04/041945.

### Sample size

This sample size was decided based on previously published studies evaluating serum and salivary IgA responses following SARS-CoV-2 infection or vaccination (Isho et al., 2020; Ketas et al., 2021). A priori sample-size estimation for a two-group comparison (independent t-test) was performed. Assuming a medium effect size (Cohen’s d = 0.5), α = 0.05, and 80% power, the minimum required total sample size was 128 participants. Our final sample size of 127 provides approximately 80% power^12,18^.

### Eligibility criteria and screening of participants for recruitment

All individuals who came to the outpatient Department of Manipal College of Dental Sciences, Manipal, and Kasturba Medical Hospital, Manipal, India, were screened based on the following criteria.

#### Inclusion criteria

Participants between the ages of 18 and 60 years who had received the two doses of the COVID-19 vaccines in India (either Covishield or Covaxin) and were willing to participate in the study were recruited after verbal and written informed consent.

#### Exclusion criteria:

- Participants who did not receive any COVID-19 vaccine or received only one dose of the COVID-19 vaccine.
- Participants who received any other vaccine except Covishield or Covaxin.
- Participants who received the second dose of vaccines two weeks before enrollment.
- Participants with untreated/ongoing infectious or any immunocompromised or autoimmune condition/disease at the time of enrolment.
- All pregnant and lactating females are to avoid any bias due to the immune response during pregnancy.
- Participants who were undergoing chemotherapy or radiation therapy.
- Participants who were receiving corticosteroids, antiviral therapy, immunomodulators, or antimicrobial agents, including antibiotics.

### Screening and data collection process

Based on the above eligibility criteria, approximately 300 individuals were screened, of which 173 participants were excluded. Out of these 173 participants, 70 participants were unwilling to participate, 98 participants had taken the 3^rd^ dose of vaccination, and 5 participants had taken vaccines two weeks before enrolment. Hence, 127 participants were included in the study after taking written informed consent. Upon enrollment, the following personal and demographic data were collected on printed data collection forms by two investigators (MS and AC): age (years); gender (female/male); address (district/city); occupation; height (cm) and weight (kg); presence/absence of systemic comorbidities (name of the disease/condition) as confirmed by medical records. The presence/absence of the following habits: smoking/gutka/paan/supari/areca nut was also noted. The name of the vaccine; presence or absence of a history of COVID-19 infection (yes/no); number of times participants have been infected with COVID-19 infection; date or month of previous infection; whether infection occurred before or after vaccination, and date/month of COVID-19 vaccination (first dose/second dose). This information was first asked verbally from all participants by the investigator based on their recall. The date/month/type of receiving the second dose of vaccination was then verified using the vaccination certificate issued by the Government of India or a confirmatory text message received by the participant at the time of vaccination. Participants’ oral hygiene status was assessed using the simplified oral hygiene index^19^.

The presence or absence of the following symptoms during the previous COVID-19 infection were recorded: cough, shortness of breath, diarrhea, constipation, fever of ≥ 100 (°F), sore throat, chills, body aches, loss of smell, loss of taste, dizziness, shortness of breath, oxygen requirement, rash, weakness, depression, and gastrointestinal (GIT) disorders. Based on these symptoms, the severity of COVID-19 infection was categorized as absent, asymptomatic, mild, and moderate according to Indian Council Medical Research (ICMR) guidelines^18^. Participants were categorized into three groups as follows: 0–6 months, 6–12 months, and > 12 months post-vaccination based on time of recruitment and last dose of COVID-19 vaccine. All participants were also grouped into two groups: controls (group 0: no prior history of COVID-19 infection) and case group (group 1: with a history of COVID-19 infection). After recording patient-related details, unstimulated saliva and blood were collected from all the participants.

### Saliva and blood collection

2–3 mL of unstimulated whole saliva was collected by using the ‘spitting method’^20^. Samples were centrifuged at 8000 rpm for 3 min, and the supernatant was collected and transferred to an Eppendorf vial. 2 mL of blood was collected from the antecubital fossa using a BD Vacutainer R (EclipseTM)^21^. The blood was then centrifuged at 3,000 rpm for 5 min to separate the serum. Both samples were stored at −80 °C until further analysis^20^.

### Estimation of IgA levels in saliva and blood serum

The saliva and serum were coded and sent for analysis to the Department of Microbiology, Kasturba Medical College , Manipal. The investigators (MM and MP) analysing the samples were blinded regarding the grouping and patient details. Saliva and serum IgA levels were measured using a human anti-2019 nCoV IgA ELISA kit (My-BioSource, USA, number: MBS7612290 COA). Saliva samples were diluted (1:50) for ELISA assay optimization according to the study by Aita et al.^22^.

### Data elements, grouping, and statistical analysis

All data was manually entered into Microsoft Excel and coded to blind the statistician. The mean salivary and serum IgA level was analyzed for individuals with and without a history of COVID-19 infection. The IgA levels were also checked based on the time of recruitment after the second dose of COVID-19 (0–6, 6–12, and > 12 months). The results were compared with regard to age (> 30 years and < 30 years), gender, history of COVID-19 infection, oral hygiene index (OHI > 1.2 & OHI < 1.2), and the presence or absence of comorbidities. Data were analyzed using SPSS version 20. The Shapiro–Wilk test was performed to check for normality. Demographic data were analyzed using the Chi-square test, Mann–Whitney U test, Kruskal–Wallis test, and post-hoc Dunn test.

## Results

### Descriptive outcomes

**Fig. 1:**
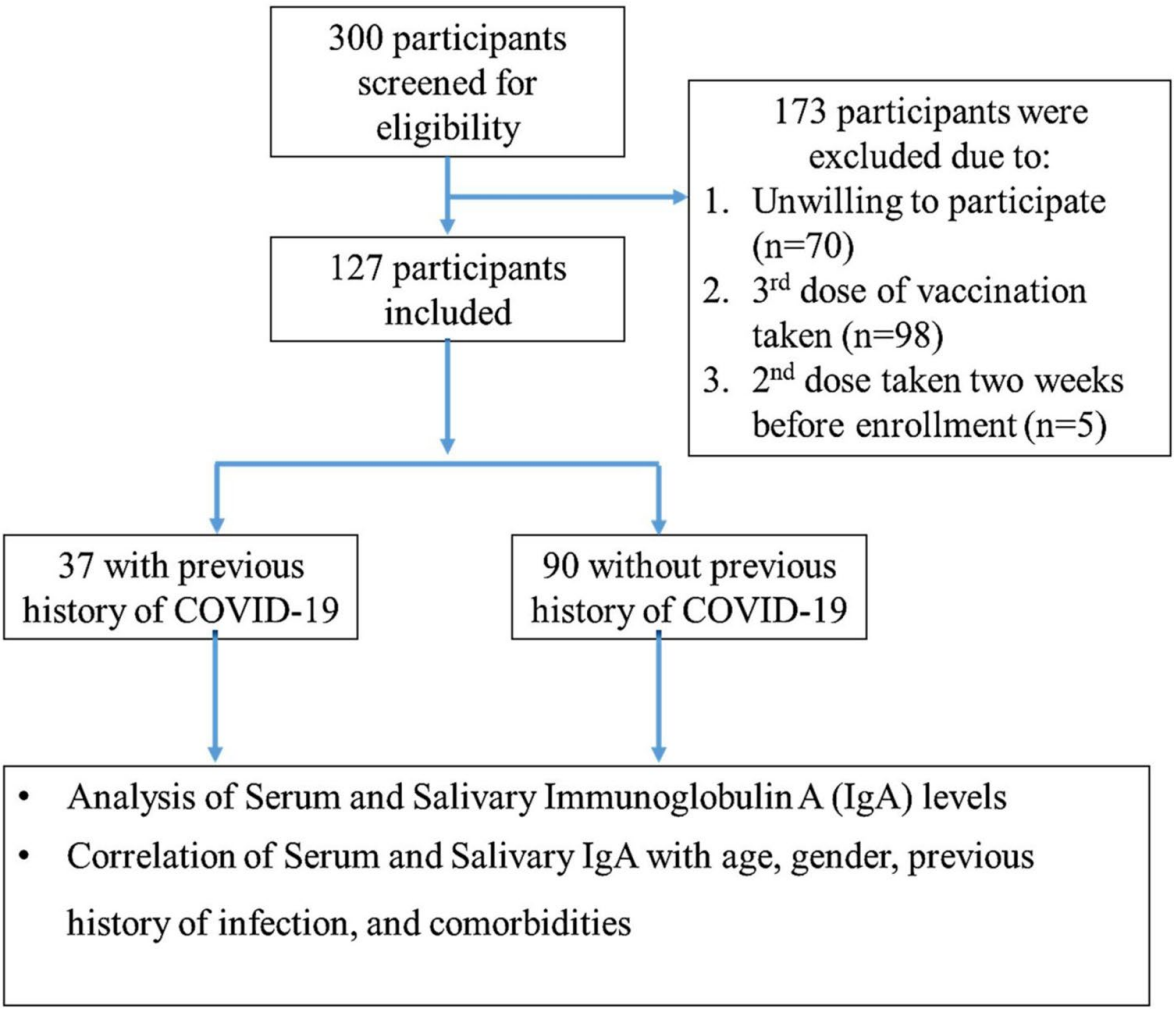
STROBE – participants flow diagram.

Of the 127 were participants, 90 (43 females and 47 males) had no history of COVID-19 infection (Group 0) and 37 (17 females and 20 males) had a history of COVID-19 infection (Group 1) (Table 1; Fig. 1). The mean age of participants in Group 0 was 31.7 years (min to max: 18–60 years), with a mean age of 31.8 years for males and a mean age of 31.5 years for females (P = 0.851). The mean age of participants in Group 1 was 27.7 years (min to max: 21–60 years), with a mean age of 27.8 years for males and 27.7 years for females (Table 1 and 2; Suppl. Table 2). Of the 127 participants, only 12 were given Covaxin and 115 were given Covisheild. Of the 127 participants, 96 (70 in Group 0 and 26 in Group 1) reported no comorbidities. Diabetes Mellitus, respiratory disease, hypertension, thyroid disease, epilepsy, and Polycystic Ovarian Disease (PCOD) were the most common comorbidities reported by all other participants (Table 1).

**Table 1:** Distribution of age, gender, number of COVID-19 infections, time between COVID-19 vaccination and COVID-19 infection, oral habits, severity, and comorbidities. P < 0.05.

Grouping criteria		Groups based on COVID-19 history					
		Without a history of COVID-19 (Group 0); N = 90		With a history of COVID-19 (Group 1); N = 37		Chi-square test	
		Count	N in %	Count	N in %	ꭓ 2 value	P value
Age (Year)	Female	43	31.5	17	27.7	−1.545	0.125
	Male	47	31.8	20	27.8		
Gender	Female	43	47.8	17	45.9	0.035	0.851
	Male	47	52.2	20	54.1		
Number of infections with COVID-19	No	90	100	0	0	127	0.001
	Once	0	0	34	91.9		
	Twice	0	0	3	8.1		
Time from COVID-19 vaccination to COVID-19 infection	No COVID-19	90	100	3	8.1	112.938	0.001
	1–3 months	0	0	4	10.8		
	4–6 months	0	0	3	8.1		
	7–9 months	0	0	13	35.1		
	10–12 months	0	0	14	37.8		
Habits	Absent	87	96.7	37	100	1.263	0.532
	Smoking	1	1.1	0	0		
	Gutkha/Paan	2	2.2	0	0		
COVID-19 infection severity	Absent	90	0	0	0	127	0.001
	Asymptomatic	0	0	5	13.5		
	Mild	0	0	28	75.7		
	Moderate	0	0	4	10.8		
Comorbidities	Diabetes	1	1.11	7	18.91	5.292	0.817
	Hypertension	3	3.33	6	16.21		
	Allergy	1	1.11	1	2.70		
	Xerostomia	0	0	0	0		
	Cardiovascular disease	0	0	2	5.40		
	Cancer	0	0	0	0		
	Kidney disorder	0	0	0	0		
	Bleeding disorder	0	0	0	0		
	High cholesterol	0	0	0	0		
	Psychological disorder	0	0	0	0		
	Respiratory disorder	3	3.33	4	10.81		
	Others	4	4.44	2	5.40		
	Obesity	0	0	1	2.70		
	Gastrointestinal disorder	1	1.11	1	2.70

**Table 2:** Mean serum IgA and saliva levels based on the interval from vaccination to sample collection (Group 1: history of COVID-19 infection; Group 0: no history of COVID-19 infection). CI of mean assumes sample means are t-distributed with N-1 degrees of freedom. P < 0.05.

	Time	Group	Mean	SD	SE	95% CI: Lower	95% CI: Upper	Median
Serum	0–6 months	1	11.22	8.80	2.933	4.46	17.99	6.80
		0	0.00	0.00	0.00	0.00	0.00	0.00
	6–12 months	1	7.95	7.77	1.089	5.76	10.14	6.30
		0	4.65	3.70	1.114	2.16	7.13	4.00
	> 12 months	1	4.18	2.87	0.658	2.80	5.57	3.40
		0	3.74	3.15	0.671	2.34	5.13	2.75
Saliva	0–6months	1	0.9000	1.9558	0.6519	−0.6033	2.4033	0.00
		0	0.0000	0.0000	0.0000	0.0000	0.0000	0.00
	6–12months	1	0.0510	0.2221	0.0311	−0.0115	0.1135	0.00
		0	0.0364	0.1206	0.0364	−0.0447	0.1174	0.00
	> 12months	1	0.0158	0.0688	0.0158	−0.0174	0.0490	0.00
		0	0.0545	0.2558	0.0545	−0.0589	0.1680	0.00

In Group 1, 8.1% (n = 3) of the participants developed COVID-19 infection before the first vaccination, and 91.9% (n = 34) developed COVID-19 infection after vaccination. Of those infected after vaccination, 83.7% of participants (n = 31) were infected once, and 8.1% of participants (n = 3) were infected twice after the second dose of COVID-19 vaccination (P = 0.001) (Table 1). The maximum number of participants were infected with COVID-19 within 6 to 12 months after the second dose of the vaccine. Within one month of vaccination, three participants acquired the Coronavirus infection. The most common post-vaccination symptoms in participants infected with COVID-19 were fever and malaise; the least common symptoms were rash, constipation, and depression (Suppl. Table 1).

### Variations in IgA levels in saliva and blood serum

A statistically significant difference in serum IgA levels was observed between individuals < 30 years of age (5.27 ± 3.14 μg/mL) and those > 30 years of age (8.93 ± 4.56 μg/mL) (P = 0.001). No statistically significant difference was found between the mean serum IgA levels in females (6.50 ± 0.21 μg/mL) and males (6.53 ± 0.21 μg/mL) (P = 0.41) (Table 2). Mean serum IgA levels were higher in Group 1 (12.59 ± 5.67 μg/mL) compared to Group 0 (8.5 ± 7.20 μg/mL).

**Fig. 2:**
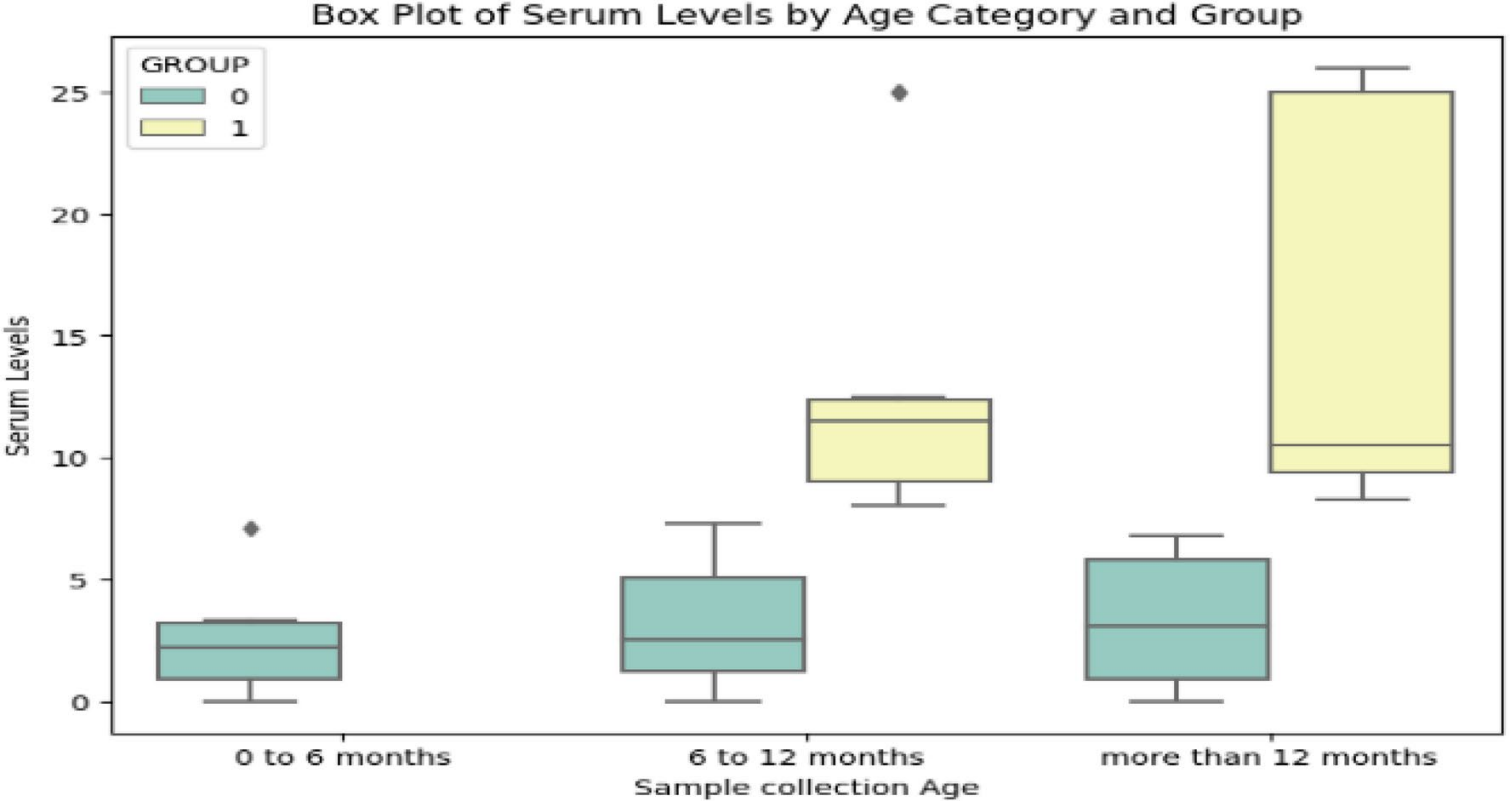
Variation in mean serum and saliva IgA levels based on time between COVID-19 vaccination and sample collection (0–6, 6–12, and > 12 months).

The mean serum IgA levels at 0–6 months, 6–12 months, and > 12 months for Group 1 were 11.22 ± 0.80 μg/mL, 7.95 ± 7.77 μg/mL, and 4.18 ± 2.87 μg/mL, respectively. The mean serum IgA levels at 0–6 months were negligible for group 0. However, the mean serum IgA levels at 6–12 months and > 12 months for Group 0 were 4.65 ± 3.70 μg/mL and 3.74 ± 3.15 μg/mL, respectively (Table 2). The mean salivary IgA level was 0.32 ± 1.9 μg/mL in Group 1 and 0.03 ± 0.37 μg/mL in Group 0. For Group 1, the mean salivary IgA level at different time intervals (within 0–6, 6–12, and > 12 months) was 0.9 ± 1.9 μg/mL, 0.05 ± 0.22 μg/mL, and 0.01 ± 0.06 μg/mL, respectively. In Group 0, no salivary IgA was detectable in participants in the 0–6 month time period. However, the salivary IgA for 6–12 months and > 12 months was 0.03 ± 0.12 μg/mL and 0.05 ± 0.25 μg/mL, respectively (Table 2, Fig. 2). The IgA levels in participants given Covishield (6.51 g/mL) and those given Covaxin were 5.86/mL. However, one must note that this may be due to the smaller number of participants given Covaxin compared to Covishield and may not be sufficient to compare the efficacy between vaccines.

### Correlation of age, gender, OHI, BMI, presence of comorbidities, and severity of COVID-19 with the mean serum IgA levels

The mean serum IgA level for participants with a BMI < 25 was 6.40 μg/mL and for those with a BMI > 25 was 6.70 μg/mL (P = 0.63) (Table 3). Based on severity, asymptomatic, mildly symptomatic, and severely symptomatic individuals showed a mean serum IgA level of 4.3 μg/mL, 4.14 μg/mL, and 2.6 μg/mL, respectively (P = 0.04) (Table 3). The serum IgA level in participants with and without comorbidities was 7.179 ± 7.59 μg/mL and 6.212 ± 6.65 μg/mL, respectively (P = 0.817). In participants with comorbidities, the group-wise comparison of mean serum IgA levels was 6.07 ± 6.35 μg/mL for Group 0 and 7.46 ± 7.09 μg/mL for Group 1 (P = 0.04). The correlation analysis of the mean serum IgA with age (< 30 and > 30), gender (female and male), OHI (< 1.2 and > 1.2), BMI (> 25 and < 25), severity of infection (asymptomatic, mild, and moderate) and comorbidities (presence/absence) showed no statistically significant correlation for all categories except for the severity of COVID-19 (Table 3).

**Table 3:** Mean serum IgA levels (in µg/mL) by age (< 30 and > 30), gender (male and female), severity (asymptomatic, mild, moderate), oral hygiene index (OHI), and body mass index (BMI). P < 0.05. SE = standard error.

Parameter				Mean ± SD			P value	SE	95^th^ Confidence interval (CI)	
									Lower limit	Upper limit
Age							0.001	0.04	0.05	0.25
< 30				5.27 ± 3.14						
> 30				8.93 ± 4.56						
Gender							0.41	1.16	−1.34	3.27
Male				6.50 ± 2.44						
Female				6.53 ± 2.13						
Severity							0.04	0.70	−2.84	−0.04
Asymptomatic				4.3 ± 1.09						
Mild				4.14 ± 1.00						
Moderate				2.6 ± 1.03						
OHI							0.53	0.73	−0.99	1.93
< 1.2				3.99 ± 1.23						
> 1.2				6.54 ± 0.33						
BMI							0.63	0.13	−0.20	0.34
< 25				6.40 ± 2.12						
> 25				6.70 ± 2.45						

Using a model-fitting approach, we found that the R^2^ value for severity was 0.155. This indicates that approximately 15.5% of the variance in the dependent variable (serum) can be explained by the independent variables in the model. The constant term has a coefficient of −0.8868 (P = 0.826). The coefficient for gender is 0.9627 (P = 0.0411), OHI 0.46 (P = 0.53), BMI 0.0067 (P = 0.631), and age −0.0238 (P = 0.002), severity −1.44 (P = 0.044).

## Discussion

Vaccinations played a crucial role in protecting and triggering the immune response against the COVID-19 infection. Vaccines help to activate the mucosal and humoral immunity and increase the levels of serum and salivary antibody (IgG and IgA) against the virus. The present study aimed to evaluate the serum and salivary IgA antibody response after COVID-19 vaccination in participants with and without a history of COVID-19 in the Udupi district of Karnataka, India. We found that COVID-19 vaccines increase the serum and salivary IgA levels following vaccination. However, the salivary IgA was detected only in those with high serum IgA levels. We also noted that those who received COVID-19 vaccines got reinfected within 6 to 12 months after vaccination. We found that the IgA antibodies were present in the serum of participants who belonged to the category of more than 12 months after vaccination. The mean serum IgA levels were higher in individuals with a previous history of COVID-19 (12.59 ± 5.67 μg/mL) compared to those without any history of COVID-19 (8.5 ± 7.20 μg/mL). Serum IgA levels were higher in those without comorbidities, those older than 30 years, and those with a history of Coronavirus infection.

We checked the levels of IgA, as it is the predominant antibody linked with mucosal immunity of the oral, nasal, and respiratory tract. The IgA antibodies are secreted in saliva and play a key role in neutralizing the toxins, enzymes, and microbes in the oral cavity. IgA also helps to maintain the integrity of the mucosal surfaces and prevents microbial adherence to mucosa, teeth, and epithelial surfaces in the nasal and oral cavity. Since the oral cavity and nose are the first and easiest portals of entry for many microbes, including Coronavirus, estimating the salivary IgA level is important to understand how effective the local antibody response is after vaccination. Individuals with good IgA antibodies in saliva will be able to neutralize the viral particles at the portal of entry itself. Several studies have shown that higher levels of mucosal sIgA correlate significantly with reduced viral load, decreased viral shedding, and lower transmission rates^7–16^. We found that the mean salivary IgA level was minimally detectable as compared to serum. The mean salivary IgA in those with previous infection was higher compared to those without infection. Although mean salivary IgA could be detected in even groups with > 12 months post-vaccination. Some reports have even stated that the efficacy of the vaccines decline by 9.0% in the first 6 to 8 months^23–26^. A study by Ketas et al. (2021) assessed the serum and salivary antibody levels (IgM, IgG, and IgA) against the S-protein and its receptor-binding domain (RBD) in healthcare workers vaccinated with Pfizer and Moderna mRNA vaccines in New York, USA. The authors observed that anti-S-protein IgG was present in 14/31 and 66/66 saliva samples from uninfected participants after the first and second doses, respectively. All serum samples from recipients of two doses of vaccine contained anti-S-protein IgG. Vaccines also induced RBD-specific antibodies, which were found frequently in saliva and serum. Although antibody levels in saliva were lower than in serum, the study emphasized that IgA plays a key role in preventing or limiting infection via the nasal and oral routes (12).

The presence of antibodies in the body and natural immunity plays a key role in determining the overall antibody response following any vaccination or reinfection. This could be attributed to the levels of antibodies formed due to the interplay between antibodies neutralized by fighting the antigen and the development of new natural or active immune responses to the Coronavirus infection. Vaccination potentially triggers and enhances the existing immune defense post-infection. Evidence also confirms that hybrid immunity, which develops in individuals who have experienced both natural SARS-CoV-2 infection and have received a COVID-19 vaccination, is stronger than immunity acquired from infection or vaccination alone. Studies have consistently shown that hybrid immunity generates higher antibody titers, stronger neutralizing activity, and more durable memory B-cell and T-cell responses^8,12,14,27^. This is because natural infection stimulates the immune system through multiple viral antigens at mucosal and systemic sites, while vaccination provides a highly targeted, high-quality boost to spike-specific responses in the immune system. Thus, the combination results in a more robust systemic IgG and IgA, as well as improved immune memory and protection against reinfection and multiple variants of the virus^28^.

A systematic review assessing the role of natural immunity after COVID-19 infection also found a high level of protection (81.0–87.0%) conferred by prior infection, which may last up to 7 months following the initial infection^23–25,29–32^. However, few studies have reported that antibodies may persist even up to 12–18 months after COVID-19 infection^33–39^. Studies have also shown that previous COVID-19 infection lowers the risk of hospitalization and death, and the antibody levels are correlated with the severity of infection^40–43^. Furthermore, there are reports that state that individuals with sustained mucosal IgA levels exhibit lower reinfection rates and reduced disease severity, underscoring the importance of IgA for long-term protective immunity^37–45^. Several studies have also assessed the antibody levels post-vaccination and infection across the globe^12,18,27,46–49^. For example, a study by Ali et al. (2021) conducted in Kuwait assessed the association between previous COVID-19 infection and the levels of IgG, IgA, and neutralizing antibodies against SARS-CoV-2 in individuals who received one or two doses of either BNT162b2 or ChAdOx1 vaccines. The author found that the mean levels of IgG, IgA, and neutralizing antibodies were higher in vaccinated subjects with previous COVID-19 infections than in those without previous infection. A steeper slope of decline for IgG and neutralizing antibodies was noted in vaccinated individuals without previous COVID-19 infection compared to those with previous COVID-19 infection^27^. Guerrieri et al. (2023) also analysed anti-SARS-CoV-2 IgA-S1 and IgG-RBD levels in serum, saliva, and nasal secretions from healthy participants without COVID-19 infection; participants vaccinated with BNT162b2 (Pfizer/BioNTech, New York, USA), and participants with a previous history of COVID-19 infection. The study found that the mRNA COVID-19 vaccine could trigger the antigen-specific mucosal immune response, producing IgA-S1 and IgG-RBD antibodies against SARS-CoV-2. This mucosal humoral response was stronger after the second vaccine dose compared to individuals recovered from COVID-19^47^. However, a recent longitudinal study by Matsumoto et al. (2024) demonstrated that antibody levels decline after booster vaccination in Japan. Prior infection enhanced post-booster immunity and maintained antibody levels for over one year. In contrast, uninfected individuals had levels that had declined by 8 months post-vaccination. Baseline post-vaccination antibodies were lower in females, the elderly, those on immunosuppressive therapy, and smokers^48^.

It is also important to note that the antibody response wanes over time, and this waning of antibodies is dependent on age, underlying medical conditions, time, type of vaccination, and previous history of infection^41–45^. A cohort study by Vinh et al. (2022) in Montreal, Canada, examined the antibody levels post-vaccination among elderly individuals over 65 years with or without a history of COVID-19 infection. The study involved 185 participants: 65 participants received two doses of mRNA-1273 (Spikevax; Moderna); 36 participants received two doses of BNT162b2 (Comirnaty; Pfizer-BioNTech), and 84 participants received a combination of mRNA-1273 followed by BNT162b2. A significant increase in the anti-RBD and anti-spike IgG levels four weeks after the initial dose was noted. This was followed by a decline until the booster dose, after which levels increased again at four weeks post-boost. Individuals without COVID-19 infection exhibited lower antibody responses than previously infected individuals at all time-points up to 16 weeks after the first dose; No differences were observed in antibody responses four weeks after the second dose between the two vaccines^46^. As compared to previous studies analysing antibodies in serum, Merking et al. (2025) reported that mucosal IgA could also be detected for at least 22 months post-infection^50^. Mades et al. assessed the development of oral SARS-CoV-2 IgG antibodies among people who received either the Moderna or Pfizer/BioNTech COVID-19 vaccination, reported no significant difference or impact of gender or age on antibody titers at 90 days after receiving the second vaccine dose^51^.

These studies confirmed that vaccinations are effective in increasing the serum and salivary IgA. However, it is crucial to note that, as compared to serum IgA levels, salivary IgA responses following vaccination are often short-lived. The rise in serum IgA post vaccination is robust and may be dependent upon previous infection^50–53^. Moreover, it should be noted that, unlike serum IgA, estimation of salivary IgA is influenced by multiple non-immunological factors such as hydration status, salivary flow rate, circadian rhythm, recent food intake, oral hygiene, and local oral conditions, including gingival inflammation or mucosal irritation. These external factors can dilute, concentrate, or alter the antibody detectability of salivary IgA and should be taken into consideration.

Since previous evidence suggests the salivary or mucosal IgA levels may not be as robust as serum IgA levels, there are some concerns that vaccinated individuals may still acquire mild or asymptomatic infections and continue to transmit the virus. This may limit or reduce the population-level effectiveness of vaccination in achieving herd immunity, which depends on preventing transmission rather than only preventing severe outcomes^54^. Thus, our goal should be to strengthen local mucosal immunity or mucosal or salivary IgA responses post-vaccination and prevent the virus at the portal of entry itself. This could be the key to achieving community-level protection. In addition, it is important to evaluate cell-mediated immune responses and how the host response differs between those with and without a history of COVID-19 infection. As IgA is found to be present in various human secretions such as serum, blood, urine, and breast milk, further studies assessing the IgA levels in breast milk of lactating females following vaccination can be done to assess whether immunity can be transferred from mothers to infants^49^. One should also note that since our study was conducted in a South Indian cohort, future studies should be conducted on a larger population across different states to include regional and environmental diversity. Future studies can compare the different innate and adaptive immune responses between different population subgroups, between different vaccinations, and also between different countries across the globe. Because the study was designed to include only vaccinated individuals, future studies can determine the role of natural immunity alone after COVID-19 infection in unvaccinated individuals and even compare it to vaccinated individuals.

## Conclusion

Serum antibody levels are increased post-vaccination. Individuals with a history of COVID-19 infection showed higher serum and salivary antibody levels. The IgA antibodies were detected in serum in the participants in the category with more than 12 months after vaccination. Salivary IgA was only detected in those with high serum IgA levels. Antibody levels were influenced by age, presence of comorbidities, and history of coronavirus infection.

## Supplementary Information

Supplementary Information.

## Data Availability

"All study data are available upon request from the corresponding author: aditi.chopra@manipal.edu. The data is also available on the preprint online: https://papers.ssrn.com/sol3/papers.cfm?abstract_id=4905383."
