# Supplementary material for: Association of COVID-19 vaccines and antibody response in individuals with prior Coronavirus infection
Source: Sci Rep. 2026 Mar 13;16:13593. doi: 10.1038/s41598-026-42177-9 (PMC13121655; doi:10.1038/s41598-026-42177-9)
Supplement: Supplementary file 1 — Supplementary Information. [file 41598_2026_42177_MOESM1_ESM.docx]

**Supplementary Table 1: Symptoms presented by participants with a history of COVID-19 (Group 1**)

| **Symptoms** | | **With COVID-19 (Group 1)** | |
| --- | --- | --- | --- |
|  |  | **Count** | **Column N %** |
| **Cough** | **No** | 19 | 51.4% |
|  | **Yes** | 18 | 48.6% |
| **Diarrhoea** | **No** | 34 | 91.9% |
|  | **Yes** | 3 | 8.1% |
| **Body ache** | **No** | 15 | 40.5% |
|  | **Yes** | 22 | 59.5% |
| **Need for oxygen** | **No** | 36 | 97.3% |
|  | **Yes** | 1 | 2.7% |
| **Loss of smell** | **No** | 29 | 78.4% |
|  | **Yes** | 8 | 21.6% |
| **Loss of taste** | **No** | 30 | 81.1% |
|  | **Yes** | 7 | 18.9% |
| **Skin rashes** | **No** | 37 | 100.0% |
|  | **Yes** | 0 | 0.0% |
| **Constipation** | **No** | 37 | 100.0% |
|  | **Yes** | 0 | 0.0% |
| **Depression** | **No** | 37 | 100.0% |
|  | **Yes** | 0 | 0.0% |
| **Weakness** | **No** | 14 | 37.8% |
|  | **Yes** | 23 | 62.2% |
| **Fever** | **No** | 14 | 37.8% |
|  | **Yes** | 23 | 62.2% |
| **Sore throat** | **No** | 17 | 45.9% |
|  | **Yes** | 20 | 54.1% |
| **Chills** | **No** | 26 | 70.3% |
|  | **Yes** | 11 | 29.7% |
| **Vertigo** | **No** | 36 | 97.3% |
|  | **Yes** | 1 | 2.7% |
| **Breathlessness** | **No** | 33 | 89.2% |
|  | **Yes** | 4 | 10.8% |
| **GIT disturbances** | **No** | 34 | 91.9% |
|  | **Yes** | 3 | 8.1% |

**Supplementary Table 2: Changes in the serum IgA levels in relation to age, weight, and height**

| **Parameter** | **Group** | **N** | **Mean** | **S.E.** | **95% confidence intervals** | | **Median** | **S.D.** | **IQR** | **Percentile** | |
| --- | --- | --- | --- | --- | --- | --- | --- | --- | --- | --- | --- |
|  |  |  |  |  | **Lower limit** | **Upper limit** |  |  |  | **25^th^** | **75^h^** |
| **Age (years)** | **0** | 90 | 31.7 | 1.38 | 28.4 | 33.9 | 25.5 | 13.10 | 15.75 | 21.0 | 36.8 |
|  | **1** | 37 | 27.7 | 1.30 | 25.1 | 30.4 | 26 | 7.91 | 5.00 | 23.0 | 28.0 |
| **Weight (kg)** | **0** | 90 | 66.07 | 1.38 | 63.3 | 68.8 | 64.0 | 13.10 | 17.00 | 58.0 | 75.0 |
|  | **1** | 37 | 68.72 | 1.81 | 65.1 | 72.4 | 66.0 | 10.99 | 13.00 | 62.0 | 75.0 |
| **Height**  **(cm)** | **0** | 90 | 165.5 | 1.13 | 163.3 | 167.8 | 164.0 | 10.70 | 15.00 | 158.0 | 173.0 |
|  | **1** | 37 | 166.4 | 1.71 | 163.0 | 169.9 | 167 | 10.43 | 12.00 | 161.0 | 173.0 |
